# *Staphylococcus ursi* sp. nov., a new member of the ‘*Staphylococcus intermedius* group’ isolated from healthy black bears

**DOI:** 10.1099/ijsem.0.004324

**Published:** 2020-07-16

**Authors:** Vincent Perreten, Stephen A. Kania, David Bemis

**Affiliations:** ^1^​ Institute of Veterinary Bacteriology, University of Bern, CH-3001 Bern, Switzerland; ^2^​ Department of Biomedical and Diagnostic Sciences, University of Tennessee College of Veterinary Medicine, Knoxville, Tennessee, USA

**Keywords:** *Staphylococcus ursi*, *Staphylococcus intermedius *group, black bear, Gram-positive, cocci, animals, *Staphylococcaceae*

## Abstract

Six *Staphylococcus* strains were isolated from healthy black bears (*Ursus americanus*) in the Great Smoky Mountains National Park, Tennessee, USA. Phylogenetic analysis based on complete genome, 16S rRNA, *dnaJ*, *hsp60*, *rpoB* and *sodA* genes, and MALDI-TOF-MS main spectral profiles revealed that the strains belonged to one species and showed the closest relatedness to members of the ‘* Staphylococcus intermedius* group’ (SIG), which include *Staphylococcus intermedius *, *Staphylococcus pseudintermedius, Staphylococcus delphini* and *Staphyloccoccus cornubiensis*. The strains were positive in SIG-specific and negative in individual species-specific PCR assays for the *nuc* gene. The strains can be differentiated from the other SIG species by the absence of sucrose fermentation, from *S. intermedius* DSM 20373^T^, *S. pseudintermedius* CCUG 49543^T^ and *S. cornubiensis* DSM 105366^T^ by the absence of methyl β-d-glucopyranoside fermentation and from *S. delphini* DSM 20771^T^ by fermentation of trehalose. DNA relatedness of the type strain MI 10-1553^T^ with the type strains of *S. delphini *, *S. pseudintermedius *, *S. intermedius* and *S. cornubiensis* was ≤48.2 % by digital DNA–DNA hybridization and ≤92.3 % by average nucleotide identity calculations. Iso-_C15:0_, anteiso-C_15 : 0_ and anteiso-C_17 : 0_ were the most common fatty acids. Polar lipids consisted of phosphadidylglycerols, phospholipids, glycolipid, diphosphatidylglycerol and aminophospholipid. Cell-wall peptidoglycan was of type A3α l-Lys-Gly_3_ (Ser; similar to A11.2 and A11.3). The respiratory quinone belonged to menaquinone 7 (MK-7). The G+C content of MI 10-1553^T^ was 39.3 mol%. The isolated strains represent a novel species of the genus *Staphylococcus *, for which we propose the name *Staphylococcus ursi* sp. nov. The type strain is MI 10-1553^T^ (=ATCC TSD-55^T^=CCOS 1900^T^).

## Introduction

Over a number of years, bacteria that share colony morphotypes and overlapping phenotypic characteristics with *Staphylococcus aureus* were taxonomically segregated and assigned to new species, e.g. *Staphylococcus intermedius* [1], *Staphylococcus delphini* [2], *Staphylococcus pseudintermedius* [3], and *Staphylococcus cornubiensis* [4]. The above species are commonly referred to as the ‘* Staphylococcus intermedius* group’ (SIG). Phenotypic traits and 16S rRNA gene sequencing do not readily differentiate between these closely related species [5]. SIG members are usually associated with various animal hosts; however, their presence in human infections may be underestimated due to the potential for misidentification as *S. aureus* [6–8]. Such misidentification can negatively affect interpretation of susceptibility test results and lead to inappropriate antimicrobial therapy [8, 9]. As with *S. aureus* in people, *S. pseudintermedius* has been well characterized as an opportunistic pathogen in dogs and the frequency of infections caused by methicillin- and multidrug-resistant strains has been increasing in recent years [10]. Overall, little is known about the host range and population distribution of species within the SIG. It has been suggested, for example, that strains identified as *S. pseudintermedius* by traditional means from hosts other than dogs should be more thoroughly investigated by phylogenetic analysis before reporting [5]. The objectives of this work were to seek SIG isolates from healthy wild bears and to confirm their species identity by polyphasic taxonomic characterization. Such strains will be useful for future comparisons to existing collections for the purpose of examining their diversity and potential to exchange virulence and resistance traits.

**Fig. 1.**
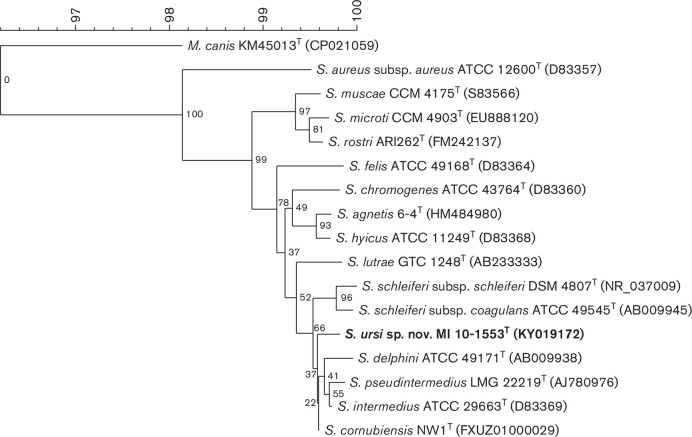
Phylogenetic tree reconstructed from 16S rRNA gene sequences of *Staphylococcus ursi* sp. nov. and the other *Staphylococcus* species of the *Staphylococcus hyicus-intermedius* species group, as well as *S. aureus *. The tree was generated by neighbour-joining analysis [Multiple alignment (OG 100 %; UG 0 %), discard unknown bases, use active zones only, without fast algorithm] and Jukes–Cantor correction using BioNumerics 7.5 (Applied Maths). Bootstrap values are shown at each node as a percentage of 1500 replicates. The 16S rRNA gene of *Macrococcus canis* KM45013^T^ was used as outgroup to root the tree. The GenBank/EMBL/DDBJ accession numbers for sequences derived from each type strain are provided in the parentheses.

During a surveillance study for the presence of staphylococci, cutaneous samples for culture were obtained from 15 healthy black bears (*Ursus americanus*) that had been captured and tranquilized for relocation, or, in one case, hit by a car and euthanized, in the Great Smoky Mountains National Park, Tennessee, USA, during 2010. All animal handling and sample collections were performed by trained and certified National Park Service wildlife biologists. Culture swabs (Becton Dickinson) were used to collect samples from the nasal cavity and a minimum of two additional pre-defined sites (nasal, oral lip folds, inguinal skin or external ear) from each animal. Swabs in Amies medium (Becton Dickinson) were kept cool while in the field and transported for processing within 24–48 h. Swabs were inoculated onto colistin–nalidixic acid agar (Becton Dickinson) containing 5 % sheep blood. The plates were incubated at 35 °C for 24–48 h. A representative of all colony types resembling those of staphylococci and that were catalase-positive, Gram-stain-positive cocci were inoculated onto trypticase soy agar containing 5 % sheep blood (TSA-SB; Becton Dickinson) and incubated at 35 °C for 24 h; subsequently, cell lysates were used as template DNA for universal bacterial 16S rRNA gene PCR followed by direct Sanger sequencing to obtain partial nucleotide sequences as described previously [11]. The 16S rRNA gene sequences of the strains were compared to sequences in the GenBank database using the blastn algorithm [12]. Strains resembling members of the SIG were further screened by partial thermonuclease gene (*nuc*) PCR using previously described group-specific [13] and species-specific [14] methods. Of the 108 strains screened, 61 were provisionally identified as belonging to the genus *Staphylococcus* and were distributed among 10 different species or groups of closely related species. Six strains, found in cultures from sample sites of five animals, had large (>5 mm), smooth, off-white colony morphologies resembling those of SIG members, displayed α hemolysis on sheep blood agar and were catalase-positive, Gram-stain-positive cocci. The strain designations and their corresponding animal, gender and sample site from which they were isolated were as follows: MI 10–1549, bear 1, female, nasal; MI 10–1553, bear 2, female, oral lip fold; MI 10–1558, bear 3, female, oral lip fold; MI 10–1605, bear 4, male, inguinal skin; MI 10–1708, bear 5, female, oral lip fold; MI 10–1710, bear 5, female, external ear canal. The six strains contained 16S rRNA gene sequences that differed from those of known *Staphylococcus* species clustering with strains of the *S. hyicus-intermedius* species group [15] and appeared to be most closely related to members of the SIG (Fig. 1). The strains could not be assigned to any of the known species of the SIG using a species-specific *nuc* PCR assay which is the currently reliable and recommended method to rapidly identify members of this group [14]. The strains were, however, positive in the SIG-specific [13] PCR assay for the *nuc* gene. These six strains were not clonally related as demonstrated by pulsed-field gel electrophoresis (PFGE; Fig. S1, available in the online version of this article) and were selected for further identification and characterization. PFGE was carried out using DNA digested with Cfr9I as described previously [16]. PFGE was run in 1 % agarose gel in 0.5×TBE on a Bio-Rad CHEF-DR III system for 18 h with pulse from 0.5 to 15 s and for 5 h with pulse from 20 to 25 s and at 14 °C and 5.6 V cm^−1^.

Whole-genome sequencing was performed for strain MI 10-1553^T^ using the MinION R.9.4.1 flow cell (Oxford Nanopore Technology), Novaseq6000 (Illumina), Ion Torrent sequencing technology (Thermo Fisher Scientific) and assembled with Unicycler (version 0.4.4). The resulting assembled sequence consisted of a 2 896 994 bp chromosome which was annotated using the NCBI Prokaryotic Genome Annotation Pipeline (GenBank acc. no. CP048279). The genome sequence was used to perform digital DNA–DNA hybridization (dDDH) and average nucleotide identity (ANI) calculations, whole genome comparative analysis, and to obtain DNA sequences for the 16S rRNA, *nuc*, *dnaJ*, *hsp60*, *rpoB* and *sodA* genes as well as the operon coding for teichoic acid biosynthesis. The 16S rRNA gene sequence of strain MI 10-1553^T^ (GenBank acc. no. KY019172) clustered into the phylogenetic branch of the SIG (Fig. 1) and shared ≤99.7 % identity to the sequence of the other SIG type strains using the lalign program [17] (Table 1). The 16S rRNA of the novel species shared 99.2 % identity with *Staphylococcus schleiferi* subsp. *coagulans* ATCC 49545^T^, which is the next closest *Staphylococcus* species to those of the SIG group, was provisionally also isolated from some of the bear samples in this study and that is commonly found in body sites occupied by *S. pseudintermedius* in dogs [10]. Specific regions of the *dnaJ* [18], *hsp60* [19], *rpoB* [20] and *sodA* [21] genes, which have been frequently used for molecular identification of staphylococci, were also amplified by PCR from the *S. ursi* strains using primers and conditions presented in Table S1 and sequenced. Alignment of these regions with those of the *S. hyicus-intermedius* group showed that the six strains were closely related but differed from those of SIG members (Table 1, Fig. S2). The DNA region of *sodA* (416 bp; GenBank acc. no. KY056143) was identical in each *S. ursi* strain and shared ≤97.6 % DNA identity to the sequence of the SIG type strains. The *hsp60* region (552 bp; GenBank acc. no. KY056141) was identical in each *S. ursi* strain and showed ≤92.9 % DNA identity to that of the SIG type strains. The DNA of the *rpoB* region (476 bp; GenBank acc. no. KY496627) shared 99.8 % between all *S. ursi* strains and ≤96.6 % DNA identity to that the SIG type strains. The DNA of the *dnaJ* region (883 bp) (GenBank acc. no. KY496628) shared 99.8 % between all *S. ursi* strains and ≤96.1 % DNA identity to that of the SIG type strains. The complete *nuc* gene of *S. ursi* MI 10-1553^T^ (GenBank acc. no. KY056142) showed ≤93.7 % identity to that of the other SIG type strains (Table 1). Since PCR assay targeting the *nuc* region is the currently recommended method to rapidly identify members of the SIG [14], specific primers *nuc-S.ursi*-F (5′-GCAGACACCTCGAAATCAATGTG) and *nuc-S.ursi*-R (5′-GGTATCCCCATCTATCACGCGT) were designed in this study to specifically amplify a 593 bp fragment of the *nuc* region of the *S. ursi* sp. nov. strains to distinguish *S. ursi* sp. nov. from the four type strains of other members of the SIG. PCR were performed using *Taq* polymerase and an initial denaturation step at 94 °C for 3 min, followed by 35 cycles of denaturation at 94 °C for 30 s, annealing at 54 °C for 30 s and elongation at 72 °C for 50 s.

**Table 1.** Percentage of identities of 16S rRNA, specific regions of *dnaJ*, *hsp60*, *rpoB*, *sodA*, digital DNA–DNA hybridization (dDDH), and average nucleotide identity (ANI) of Staphylococcus ursi MI 10-1553^T^ as compared with the other type strains of the *Staphylococcus intermedius* group (SIG) The ANI were calculated using OrthoANIu [24], and the dDDH was determined using the Genome-to-Genome Distance Calculator (GGDC) [23]. Identity between the genetic markers was determined using the lalign program [17]; nd, No cut off defined; CI, confidential interval.

Type strains (GenBank accession no. of the genome)	Genetic features and cut off values [references]							
	16S rRNA gene (98.7 %) [28]	*dnaJ* (883 bp region) (88.8 %) [18]	*hsp60* (552 bp region) (93 %) [19]	*rpoB* (476 bp region) (93.6 %) [20]	*sodA* (416 bp region) (97 %) [21]	*nuc* (507 bp) (ND)	dDDH (70 %) [28]	ANI (95–96 %) [28]
* S. pseudintermedius* LMG 22219^T^ (MLGE02000000)	99.6	95.0	92.4	96.0	96.6	93.5	46.5 [CI 43.9–49.1 %]	91.99
* S. intermedius* NCTC 11048^T^ (UHDP00000000)	99.7	92.4	91.7	95.0	91.6	89.2	36.6 [CI 34.1–39.1 %]	88.84
* S. cornubiensis* NW1^T^ (FXUZ00000000)	99.7	91.3	92.9	92.9	95.9	86.4	34.7 [CI 32.2–37.2 %]	87.95
* S. delphini* NCTC 12225^T^ (LR134263)	99.4	96.1	92.2	96.6	97.6	78.7	48.20 [CI 45.6–50.8 %]	92.32

The genome sequences were uploaded to the Type (Strain) Genome Server (TYGS) hosted at the Leibniz Institute DSMZ, Germany (https://tygs.dsmz.de) for whole-genome comparative analysis and dDDH calculations [22]. All pairwise comparisons among the set of genomes were conducted using genome blast distance phylogeny (GBDP) and intergenomic distances inferred under the algorithm 'trimming' and distance formula d5 with a 100 distance replicates calculation [23]. The dDDH values and confidence intervals were calculated using the recommended settings and formula d4 of the Genome-to-Genome Distance Calculator (GGDC version 2.1) [23]. The ANI value of the genome of *S. ursi* MI 10-1553^T^ as compared to the other SIG type strains has been calculated using orthoANIu [24].

**Fig. 2.**
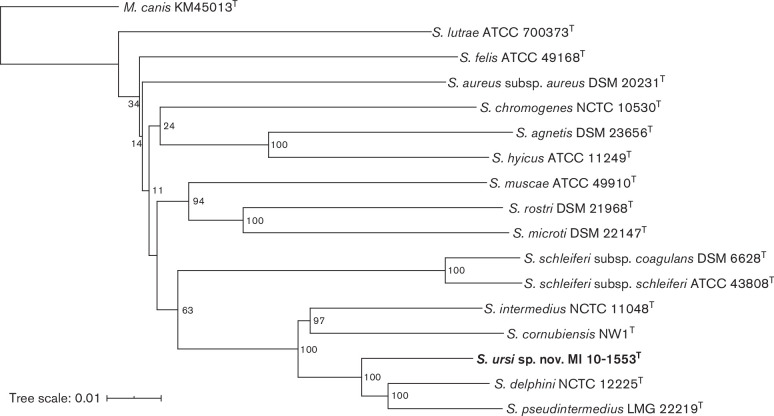
Balanced minimum-evolution tree containing strain MI 10-1553^T^ and the other type strains of the *Staphylococcus hyicus-intermedius* group, as well as *S. aureus *. The tree was generated using the Type (Strain) Genome Server (TYGS) (https://tygs.dsmz.de) including the following whole genome sequences: *S. aureus* subsp. *aureus* DSM 20231^T^ (NZ_CP011526.1), *S. muscae* ATCC 49910^T^ (NZ_CP027848.1), *S. microti* DSM 22147^T^ (JXWY01000001-JXWY01000186), *S. rostri* DSM 21968^T^ (PPRF01000001–PPRF01000167), *S. felis* ATCC 49168^T^ (NZ_CP027770), *S. chromogenes* NCTC10530^T^ (UHDB01000001–UHDB01000002), *S. agnetis* DSM 23656^T^ (PPQF01000001–PPQF01000164), *S. hyicus* ATCC 11249^T^ NZ_CP008747), *S. lutrae* ATCC 700373^T^ (NZ_CP020773.1), *S. schleiferi* subsp. *coagulans* DSM 6628^T^ (PPQN01000001–PPQN01000155), *S. schleiferi* subsp. *schleiferi* ATCC 43808^T^ (POVK01000001–POVK01000088), *S. delphini* NCTC12225^T^ (NZ_LR134263), *S. pseudintermedius* LMG 22219^T^ (MLGE02000001–MLGE02000036), *S. intermedius* NCTC 11048^T^ (UHDP01000001–UHDP01000003), *S. cornubiensis* NW1^T^ (FXUZ01000001–FXUZ01000136), *S. ursi* sp. nov. MI 10-1553^T^ (CP048279) and *Macrococcus canis* strain KM45013^T^ (NZ_CP021059). The tree was reconstructed using the intergenomic distances obtained from the GBDP analysis and with branch support (100 pseudo-bootstrap replicates) via fastme 2.1.4 and including subtree pruning and regrafting postprocessing [25]. The genome of *M. canis* KM45013^T^ was used as outgroup to root the tree. The tree was visualized and rooted using iTOL (https://itol.embl.de/) [26]

A balanced minimum evolution tree of strain MI 10-1553^T^ and its next-related *Staphylococcus* species was reconstructed using the intergenomic distances obtained from the GBDP analysis and with branch support (100 pseudo-bootstrap replicates) via fastme 2.1.4 and including subtree pruning and regrafting postprocessing [25]. The tree was visualized using iTOL (https://itol.embl.de/) [26] (Fig. 2). Phylogenetic relationships of the novel strains and other staphylococcal species were also determined for 16S rRNA gene, *sodA*, *hsp60*, *dnaJ* and *rpoB*, and analysing sequences using BioNumerics 7.5 (Applied Maths). The trees were generated by the neighbour-joining method [multiple alignment (OG 100 %; UG 0 %), discard unknown bases, use active zones only] using the Jukes–Cantor correction. Bootstrap values were determined from 1500 replications. The phylogenetic trees showed that the novel strains, as represented by strain MI 10-1553^T^, clustered within the *S. hyicus-S. intermedius* group [15] and are most closely related to the SIG (Figs 1, 2 and S2).

Strain MI 10-1553^T^ also differed from the type strains of species within the SIG by dDDH and ANI analyses. A threshold value of 70 % DNA–DNA relatedness and of 95–96 % ANI for the definition of bacterial species was considered as recommended [27, 28]. Strain MI 10-1553^T^ did not belong to known species within the SIG with dDDH values ≤48.20 % and with ANI values ≤48.2 % with the genome of *S. delphini* NCTC 12225^T^ (=DSM 20771^T^), *S. pseudintermedius* LMG 22219^T^ (=CCUG 49543^T^), *S. intermedius* NCTC 11048^T^ (=CCUG 20373^T^), and *S. cornubiensis* NW1^T^ (=DSM 105366^T^), which were the closest relatives by 16S rRNA, *dnaJ*, *hsp60*, *rpoB*, *sodA* and whole genome sequence comparison (Table 1).

The DNA G+C content of strain MI 10-1553^T^ was 39.3 mol%, as derived from the complete genome sequence. The G+C contents are within the range of 33–40 mol% reported for other *Staphylococcus* species [29]. Menaquinone profile, peptidoglycan structure, polar lipids and fatty acid composition were determined at the Leibniz Institute DSMZ-German Collection of Microorganisms and Cell Cultures, Germany. Fatty acid analysis was performed by gas chromatography (www.midi-inc.com). Fatty acid methyl esters were obtained from 40 mg cells scraped from trypticase soy broth agar plates incubated at 28 °C for 24 h by saponification, methylation and extraction using minor modifications of the method of Miller [30] and Kuykendall *et al*. [31]. The fatty acid methyl esters mixtures are separated using Sherlock Microbial Identification System (MIS; midi, Microbial ID) which consisted of an Agilent model 6890 N gas chromatograph fitted with a 5 % phenyl-methyl silicone capillary column (0.2 mm×25 m), a flame ionization detector, Agilent model 7683A automatic sampler and an HP-computer with the midi database (Hewlett-Packard). Peaks are automatically integrated and fatty acid names and percentages calculated by the MIS Standard Software (Microbial ID). The gas chromatographic parameters are as follows: carrier gas, ultra-high-purity hydrogen; column head pressure 60 kPa; injection volume 2 µl; column split ratio, 100 : 1; septum purge 5 ml min^−1^; column temperature, 170–270 °C at 5 °C min^−1^; injection port temperature, 240 °C; and detector temperature, 300 °C. Fatty acids iso-C_15 : 0_, anteiso-C_15 : 0_ and anteiso-C_17 : 0_ were the most abundant fatty acids in strain MI 10-1553^T^. With the exception of anteiso-C_17 : 0_, these fatty acids were also the most common in the other species of the SIG (Table 2).

**Table 2.** Fatty acid composition of strain MI 10-1553^T^ and other species of the *Staphylococcus intermedius* group Strains: 1, MI 10-1553^T^; 2, *Staphylococcus intermedius* DSM 20373^T^; 3, *Staphylococcus pseudintermedius* CCUG 49543^T^; 4, *Staphylococcus delphini* DSM 20771^T^; 5, *Staphylococcus cornubiensis* DSM 105366^T^. Values are percentages of total fatty acids. Major components (>10 %) are highlighted in bold. –, Not detected.

Fatty acid	1	2	3	4	5
iso-C_13 : 0_	0.6	0.5	–	0.6	–
iso-C_14 : 0_	0.5	0.6	–	0.5	–
C_14 : 0_	0.6	0.6	–	0.9	–
iso-C_15 : 0_	**35.0**	**54.2**	**47.9**	**53.9**	**47.1**
anteiso-C_15 : 0_	**31.5**	**22.2**	**17.2**	**30.4**	**18.8**
iso-C_16 : 0_	1.1	1.3	1.3	0.9	1.8
C_16 : 0_	1.4	1.8	2.0	1.9	2.1
iso-C_17 : 0_	9.4	7.9	**11.7**	5.9	**13.9**
anteiso-C_17 : 0_	**13.6**	3.0	5.6	3.8	5.9
C_18 : 2_ ω6,9*c*/anteiso-C_18 : 0_	–	3.0	4.3	1.3	–
C_18 : 1_ ω9*c*	–	–	1.3	–	–
C_18 : 0_	1.6	2.5	2.5	–	2.6
iso-C_19 : 0_	1.1	0.7	1.1	–	2.9
anteiso-C_19 : 0_	1.6	–	0.4	–	–
C_20 : 2_ ω6,9*c*	–	0.6	3.3	–	–
C_20 : 0_	2.0	0.7	1.5	–	5.0
Unidentified	–	0.4	–	–	–

The peptidoglycan structure of strain MI 10-1553^T^ was determined as previously described using cells grown in brain heart infusion (BHI) broth (BBL, Becton Dickinson) at 37 °C for 18 h [32]. Total hydrolysates (4 N HCl, 16 h, 100^o^ C) of the peptidoglycan in strain MI 10-1553^T^ contained the amino acids Lys, Ala, Ser, Gly and Glu in molar ratios of 0.9, 1.4, 0.3, 2.8, and 1.0, respectively. The identity of amino acids was confirmed by GC/MS (GC/MS 320 Singlequad, Varian). Partial hydrolysates of the peptidoglycan (4 N HCl, 0.75 h at 100 °C), determined by 2D-TLC, revealed the presence of peptides l-Ala-d-Glu, Ala-Gly, l-Lys-Gly, d-Ala-l-Lys-Gly, oligo-Gly. The peptidoglycan type of MI 10-1553^T^ was A3α l-Lys-Gly_3_(Ser) and was similar to types A11.2 and A11.3 according to the system proposed by Schleifer and Kandler [33] for the characterization and representation of peptidoglycan types (www.peptidoglycan-types.info). The presence of serine and a reduced amount of glycine suggests that glycine is partially substituted by serine in the interpeptide bridge.

Respiratory lipoquinones and polar lipids were extracted from 100 mg freeze-dried cell material from a 24 h culture in a trypticase soy yeast extract medium at 37 °C using a the two-stage method described by Tindall [34, 35]. Respiratory lipoquinones were extracted first followed by the polar lipids [34, 35]. Respiratory lipoquinones were separated into their different classes (menaquinones and ubiquinones) by TLC on silica gel (Macherey-Nagel art. no. 805 023), using hexane/tert-butyl methyl ether (9; 1, v/v) as solvent. UV-absorbing bands corresponding to menaquinones or ubiquinones were removed from the plate and analysed further by HPLC. This step was carried out on an LDC Analytical HPLC (Thermo Separation Products) fitted with a reversed-phase column (Machery-Nagel; 2×125 mm, 3 µm, RP18) using methanol as the eluent. Respiratory lipoquinones were detected at 269 nm. Strain MI 10-1553^T^ contained menaquinone 7 (MK 7).

Polar lipids were separated by two-dimensional silica gel TLC (Macherey-Nagel, Art. No. 818 135). The first direction was developed in chloroform–methanol–water (65 : 25 : 4, v/v/v), and the second in chloroform–methanol–acetic acid–water (80 : 12 : 15 : 4, v/v/v/v). Total lipid material was detected using molybdatophosphoric acid and specific functional groups detected using spray reagents specific for defined functional groups [36]. Polar lipids of strain MI 10-1553^T^ consisted of phosphadidylglycerol, diphosphatidylglycerol, two unknown aminophospholipids, two unknown phospholipids, two unknown glycolipids and two unknown lipids (Fig. S3).

The presence of teichoic acid in the novel species was determined by the detection within the genome of strain MI 10-1553^T^ of the Tar/Tag operon (TagAHGBD) which is involved in the cell-wall teichoic acid biosynthesis [37].

No known antibiotic resistance and virulence genes were detected within the genome sequence of strain 10-1553^T^ (GenBank acc. no. CP048279) using The Comprehensive Antibiotic Resistance Database (Card) (https://card.mcmaster.ca) and the Virulence Factor Database (VFDB) (www.mgc.ac.cn/VFs/main.htm).

Matrix-assisted laser desorption/ionization time-of-flight mass spectroscopy (MALDI-TOF-MS) was performed from colonies grown on TSA-SB agar plates for 18 h at 37 °C using the ethanol–formic acid extraction method for better resolution and following the manufacturer’s instructions (Application Note, MT-80, Microflex LT, Bruker Daltonics). Each of the strains generated similar ribosomal protein spectra, but no reliable identification was obtained using the manufacturer’s database. Following manual inclusion of spectra of strain MI 10-1553^T^ to the database, all the novel bear strains were re-analysed and matched to *S. ursi* with score above 2.2, whereas the next closest species was *S. delphini* with scores underneath 1.8 (Fig. S4). It was determined that the bear strains had specific ribosomal protein spectra which were not related to any of those of the *Staphylococcus* species already represented in the manufacturer’s database at the time of testing.

Phenotypic characterization of the novel bear strains and reference strains *S. intermedius* (DSM 20373^T^), *Staphylococcus cornubiensis* DSM 105366^T^, *S. delphini* (DSM 20771^T^) and *S. pseudintermedius* (CCUG 49543^T^) was initially tested using both Vitek 2 GP card (bioMérieux) and API ID 32 Staph V3.0 (bioMérieux) system following the manufacturer’s recommendations. Results obtained with Vitek 2 system (bioMérieux) were confirmed for overlapping tests with the API ID 32 STAPH system (bioMérieux) (Table 3). None of the additional tests present in the API ID 32 STAPH kit distinguished *S. ursi* from the other SIG strains. *S. ursi* sp. nov. can be differentiated from *S. intermedius *, *S. pseudintermedius *, *S. delphini* and *S. cornubiensis* by absence of acid production from sucrose; however, many strains of *S. delphini* showed weak reactions with sucrose and were considered as negative by Sasaki *et al*. [38]. The bear strains were differentiated from *S. delphini* (DSM 20771^T^) by acid production from trehalose; however, 94 % of 17 genotypically confirmed field strains of *S. delphini* Group A [38] also produced acid from trehalose. The *S. ursi* sp. nov. strains differed from *S. intermedius *, *S. pseudintermedius* and *S. cornubiensis* by acid production from methyl β-d-glucopyranoside. Absence of acetoin production was confirmed by the Voges–Proskauer test as described by Barritt [39]. Catalase activity was determined by direct application of cells from a colony to a drop of 3 % H_2_O_2_ on a glass slide. DNAase activity was tested on DNAase Test Agar (BBL, Becton Dickinson). Thermonuclease activity was determined by spotting 20 ml filtered overnight culture supernatant on DNase test agar (BBL, Becton Dickinson) subsequently incubated for 2 h at 60 °C and flooded with 1 M HCl. Using this method, all bear strains tested negative for thermonuclease; the type strains of *S. intermedius *, *S. pseudintermedius* and *S. delphini* were positive. Coagulase was determined using both a rabbit plasma tube (BBL, Becton Dickinson) and slide tests (Sigma-Aldrich). Cytochrome oxidase was tested using Microdase discs (Remel). Growth assays with different salt concentrations (6.5, 9.0 and 12 .0%, w/v NaCl) and at 18, 35 and 43 °C were performed in BHI broth. Anaerobic growth was tested in thioglycolate and on blood agar plates incubated at 35 °C in an atmosphere of 5 % hydrogen, 10 % CO_2_ and 85 % nitrogen. The Christie–Atkins–Munch–Petersen (CAMP) reaction was determined with *Streptococcus agalactiae* strain ATCC 12386 on Columbia Agar with 5 % sheep blood as previously described [40]. Lysostaphin susceptibility was determined by examining zones of growth inhibition surrounding discs saturated with 25 µg of lysostaphin after incubation on Mueller–Hinton agar (BBL, Becton Dickinson) overnight at 37 °C.

**Table 3.** Biochemical characteristics of the novel strains (Staphylococcus ursi sp. nov.) and the reference strains of the *Staphylococcus intermedius* group (SIG) Strains: 1, MI 10–1549; 2, MI 10-1553^T^; 3, MI 10–1558; 4, MI 10–1605; 5, MI 10–1708; 6, MI 10–1710; 7, *Staphylococcus intermedius* DSM 20373^T^; 8, *Staphylococcus pseudintermedius* CCUG 49543^T^; 9, *Staphylococcus delphini* DSM 20771^T^; 10, *Staphylococcus cornubiensis* DSM 105366^T^. All data are from this study. +, Positive; −, negative; (+), weakly positive; (−), weakly negative.

Characteristics	*Staphylococcus ursi* sp. nov. (1 to 6)						SIG (7 to 10)			
	1	2	3	4	5	6	7	8	9	10
Alanine arylamidase	−	−	−	−	−	−	+	+	+	−
Leucin arylamidase	−	(+)	−	−	−	−	−	+	+	+
l-Pyrrolidonyl-arylamidase	+	+	+	−	+	(+)	+	+	+	+
l-Lactate alkalinization	+	+	+	+	−	+	+	+	+	+
Methyl β-d-glucopyranoside	−	−	−	−	−	−	+	+	−	+
*N*-Acetyl-d-glucosamine	+	+	−	+	+	+	−	+	+	+
Urease	+	+	+	+	−	+	+	+	+	−
α-Glucosidase	−	(−)	−	−	−	−	+	+	−	+
α-Galactosidase	(−)	+	−	−	−	−	−	−	−	−
Bacitracin resistance	−	(−)	−	−	−	−	+	+	−	−
Acid production from:										
d-Ribose	+	+	−	+	+	+	+	+	+	+
d-Galactose	+	+	+	+	+	+	−	+	−	−
Maltose	+	+	−	−	+	+	+	+	+	−
d-Mannitol	+	−	+	+	(−)	+	+	−	−	+
d-Mannose	−	+	−	(−)	−	+	+	+	+	+
Sucrose	−	−	−	−	−	−	+	+	+	+
Trehalose	+	+	+	+	+	+	+	+	−	+

In addition to their similarities to *S. aureus* and members of the SIG, colony phenotypes of the bear strains on TSA-SB (large, smooth, off-white with alpha hemolysis) also resembled those described for *S. schleiferi* [41]. In this study the novel bear strains were phylogenetically most closely related to members of the SIG and *S. schleiferi* occupied the next closest clade (Figs. 1 and 2). The novel bear strains can be differentiated from *S. schleiferi* by the absence of acetoin production.

Based on the combined phenotypic and genotypic characteristics of the bear strains, these strains, represented by strain MI 10-1553^T^, meet criteria for being assigned to a new *Staphylococcus* species, for which we propose the name *Staphylococcus ursi* sp. nov.

## Description of *Staphylococcus ursi* sp. nov.

*Staphylococcus ursi* (ur'si. L. gen. n. *ursi* of a bear).

Cells are non-motile, Gram-positive cocci (0.5–1.5 µm). Well-isolated colonies are up to 2 mm in diameter after 24 h incubation at 35 °C and exceed 5 mm in diameter after 5 days of growth. Colonies appear convex, smooth, off-white, lack carotenoid pigment and display α haemolysis on sheep blood agar. Strains have a positive CAMP reaction with *Streptococcus agalactiae *, indicating production of δ-toxin, except strain MI 10 1558. Grows aerobically and anaerobically. Grows in 6.5, 9 and 12% NaCl (w/v) and at 43 and 18 °C. Catalase-positive, oxidase-negative and coagulase-negative. Produces thermostable nuclease with zones of hydrolysis up to 5 mm. Positive for: arginine dihydrolase 1 (arginine dihydrolase 1 and arginine dihydrolase 2 are separate proprietary tests, each formulated to provide differentiation to the species claimed on the manufacturer’s identification card), β-galactosidase, alkaline phosphatase, optochin resistance, O/129 resistance and acid production from glucose, fructose, lactose, trehalose and d-galactose. Negative for: arginine dihydrolase 2, ornithine decarboxylase, aesculin hydrolysis, acetoin production, arginine arylamidase, β-glucuronidase, α-mannosidase, l-aspartate arylamidase, alanine-phenylalanine-proline arylamidase, l-proline arylamidase, tyrosine arylamidase, phosphatidylinositol-phospholipase C, cyclodextrin utilization and acid production from raffinose, cellobiose, sucrose, turanose, arabinose, xylose, sorbitol, salicin, methyl β-d-glucopyranoside, β-galactopyranoside, pullulan and d-amygdalin. Variable reactions for: pyrrolidonyl arylamidase, leucine arylamidase, l-lactate alkalinization, α-galactosidase, α-glucosidase, urease and acid production from mannose, maltose, mannitol, ribose and *N*-acetyl-glucosamine. Susceptible to novobiocin and lysostaphin. Susceptible to polymyxin B and variable susceptibility to bacitracin.

The type strain is MI 10-1553^T^ (=ATCC TSD-55^T^=CCOS 1900^T^). This strain was isolated from the oral lip folds of a healthy black bear. The GenBank/EMBL/DDBJ accession numbers for the 16S rRNA, *hsp60*, *nuc*, *sodA, tagAHGBD*, *dnaJ* and *rpoB* gene sequences of strain MI 10-1553^T^ are KY019172, KY056141, KY056142, KY056143, KY056144, KY496628 and KY496627, respectively. The complete genome sequence of strain MI 10-1553^T^ has been deposited into GenBank under accession numbers CP048279. The associated BioProject and BioSample accession numbers are PRJNA602989 and SAMN13915443, respectively. The raw reads were deposited into the SRA database with accession numbers SRR10973935 (Illumina) and SRR10973936 (ONT).

## Supplementary Data

Supplementary material 1
