# Supplementary material for: Staphylococcus ursi sp. nov., a new member of the ‘Staphylococcus intermedius group’ isolated from healthy black bears
Source: Int J Syst Evol Microbiol. 2020 Jul 16;70(8):4637–45. doi: 10.1099/ijsem.0.004324 (PMC7660245; doi:10.1099/ijsem.0.004324)
Supplement: Supplementary material 1 [file ijsem-70-4637-s001.pdf]

Staphylococcus ursi **sp. nov., a new member of the '*Staphylococcus intermedius Group*' isolated from healthy black bears**

Vincent Perreten[1], Stephen A. Kania[2*], David A. Bemis[2]

[1]Institute of Veterinary Bacteriology, University of Bern, Bern, Switzerland;

[2]Department of Biomedical and Diagnostic Sciences, University of Tennessee College of Veterinary Medicine, Knoxville, Tennessee, United States

***Corresponding author.** Mailing address: Department of Biomedical and Diagnostic Sciences, University of Tennessee College of Veterinary Medicine, 2407 River Drive, Knoxville, TN 37996.

E-mail: skania@utk.edu

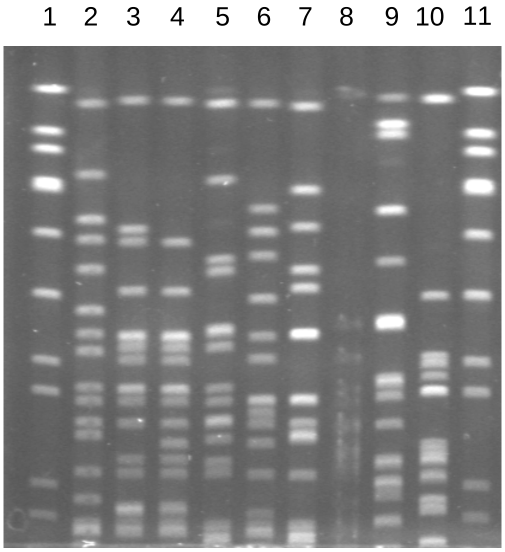

Supplementary Figure S1. Pulsed-field gel electrophoresis (PFGE) of Cfr9I-digested DNA from *Staphyloccocus ursi* sp. nov. (lanes 2 to 7) and other type strains of the *Staphyloccocus intermedius* group, namely *Staphyloccocus delphini* (lane 8), *Staphyloccocus intermedius* (lane 9) and *Staphyloccocus pseudintermedius* (lane 10). 1 and 11, *Staphyloccocus aureus* NCTC 8325 as marker; 2, MI 10-1549; 3, MI 10-1553^{T}; 4, MI 10-1558; 5, MI 10-1605; 6, MI 10-1708; 7, MI 10-1710; 8, DSM 20771^{T}; 9, DSM 20373^{T}; 10, CCUG 49543^{T}. PFGE was run in 1% agarose gel in 0.5X TBE on a Bio-Rad CHEF-DR III system for 18h with pulse from 0.5 to 15s and for 5h with pulse from 20 to 25s and at 14°C and 5.6 V/cm.

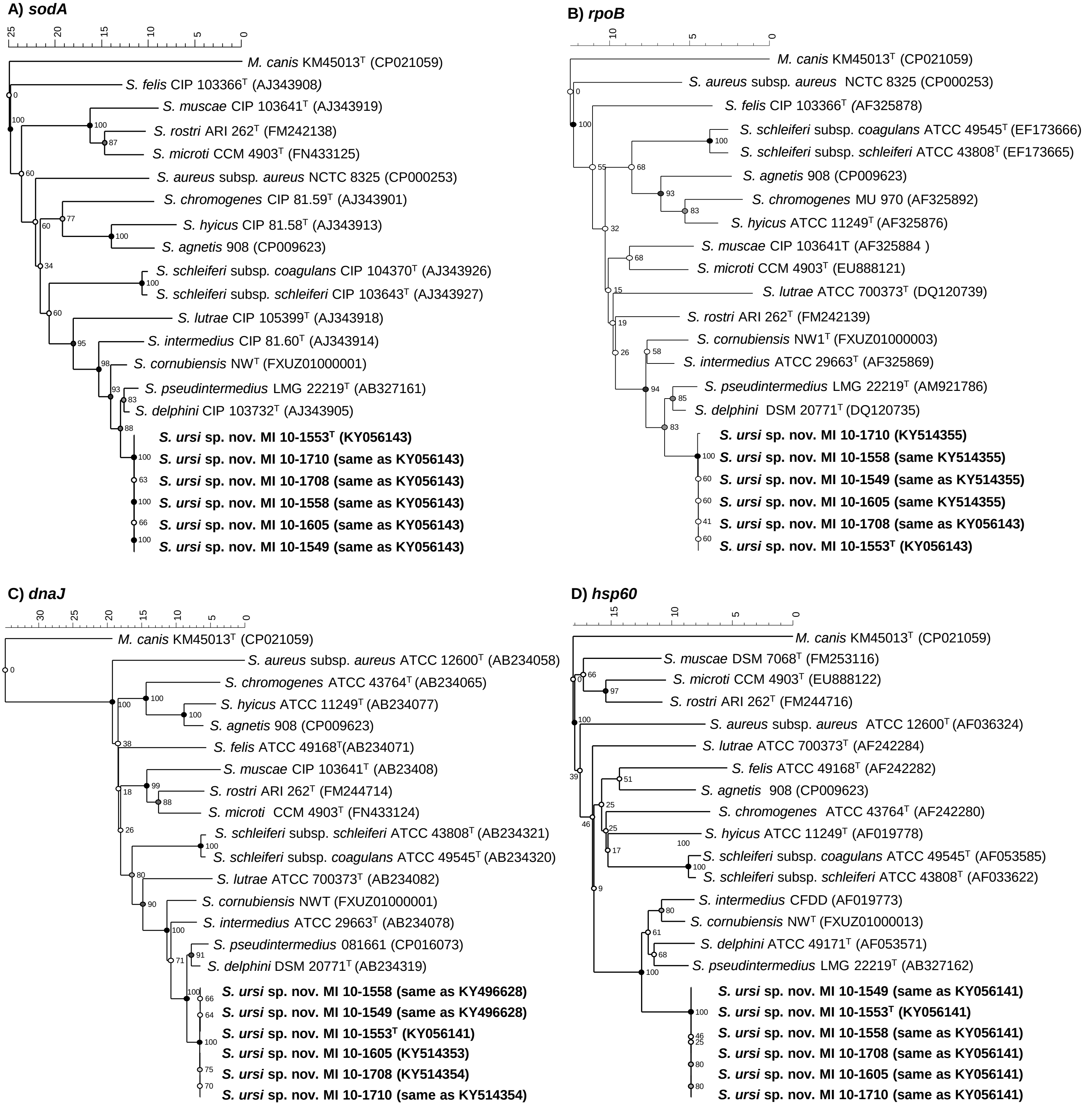

Supplementary Figure S2: Phylogenetic tree constructed from partial gene sequences of *sodA* (416 bp) **(A)**, *rpoB* (476 bp) **(B)**, *dnaJ* (883 bp) **(C)**, and *hsp60* (552 bp) **(D)** of *Staphylococcus ursi* sp. nov. and the *Staphylococcus* species of the *Staphylococcus hyicus-intermedius* group, as well as *S. aureus*. The trees were generated by Neighbor Joining analysis [Multiple alignment (OG: 100%, UG: 0%), discard unknown bases, use active zones only] and Jukes-Cantor correction using BioNumerics 7.5 (Applied Maths, Sint-Martens-Latem, Belgium). Bootstrap values are shown at each node as percentage of 1500 replicates. The sequences of the *Macrococcus canis* type strain KM45013^{T} were used as outgroup to root the trees. The GenBank/EMBL/DDBJ accession numbers are provided in the parentheses.

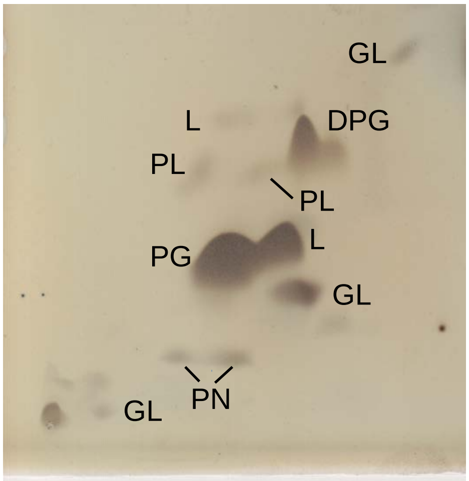

Supplementary Figure S3. Polar lipid profiles of *Staphylococcus ursi* MI 10-1553^{T} as determined by two-dimensional thin-layer chromatography. L, lipid; PL, phospholipid; PG, phosphatidylglycerol; PN, aminophospholipid; GL, glycolipid; DPG, diphosphatidylglycerol.

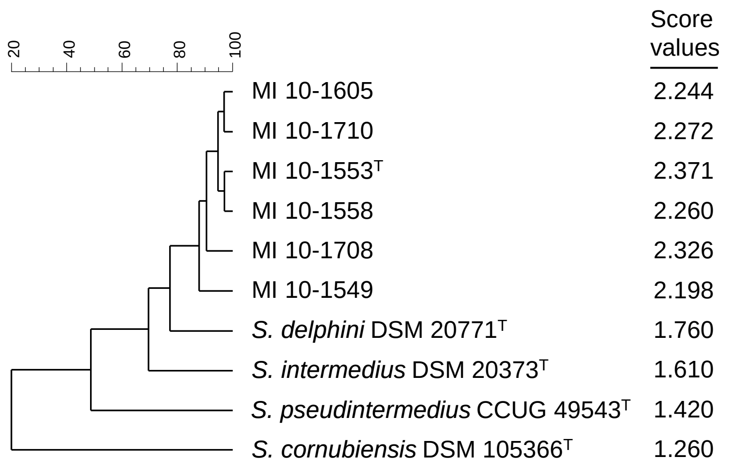

Supplementary Figure S4. Dendrogram obtained by cluster analysis of MALDI-TOF MS (Bruker Biotyper version 3.1) spectrum profiles (SP) of 6 strains of *Staphylococcus ursi* sp. nov. including type strain *S. ursi* MI 10-1553^{T} and of the other type strains of the *Staphylococcus intermedius* group (SIG), namely *S. intermedius, S. pseudintermedius* and *S. delphini.* Each SP was obtained from 3 different measurements. The score values were obtained using the MSP of *S. ursi* MI 10-1553^{T} as reference. The tree was constructed using Pearson's correlation with UPGMA clustering using Bionumerics 7.6 (Applied Maths, Sint-Martens-Latem, Belgium).
